# The G-Protein-Coupled Membrane Estrogen Receptor Is Present in Horse Cryptorchid Testes and Mediates Downstream Pathways

**DOI:** 10.3390/ijms22137131

**Published:** 2021-07-01

**Authors:** Maciej Witkowski, Laura Pardyak, Piotr Pawlicki, Anna Galuszka, Magdalena Profaska-Szymik, Bartosz J. Plachno, Samuel Kantor, Michal Duliban, Malgorzata Kotula-Balak

**Affiliations:** 1 University Centre of Veterinary Medicine JU-UA, University of Agriculture in Krakow, Mickiewicza 24/28, 30-059 Krakow, Poland; maciej.witkowski@urk.edu.pl (M.W.); anna.galuszka@urk.edu.pl (A.G.); magdalena.profaska-szymik@urk.edu.pl (M.P.-S.); samuel.kantor@urk.edu.pl (S.K.); 2 Equine Hospital on the Racing Truck, Sluzewiec, Pulawska 266, 02-684 Warszawa, Poland; 3 Center for Experimental and Innovative Medicine, University of Agriculture in Krakow, Redzina 1c, 30-248 Krakow, Poland; laura.pardyak@urk.edu.pl (L.P.); piotr.pawlicki@urk.edu.pl (P.P.); 4 Department of Plant Cytology and Embryology, Institute of Botany, Jagiellonian University in Krakow, Gronostajowa 9, 30-387 Krakow, Poland; bartosz.plachno@uj.edu.pl; 5 Department of Endocrinology, Institute of Zoology and Biomedical Research, Jagiellonian University in Krakow, Gronostajowa 9, 30-387 Krakow, Poland; michal.duliban@doctoral.uj.edu.pl

**Keywords:** cryptorchidism, estrogens, G protein-coupled receptor, horse

## Abstract

Cryptorchidism in horses is a commonly occurring malformation. The molecular basis of this pathology is not fully known. In addition, the origins of high intratesticular estrogen levels in horses remain obscure. In order to investigate the role of the G-protein-coupled membrane estrogen receptor (GPER) and establish histological and biochemical cryptorchid testis status, healthy and cryptorchid horse testes were subjected to scanning electron microscopy analysis, histochemical staining for total protein (with naphthol blue black; NBB), acid content (with toluidine blue O; TBO), and polysaccharide content (with periodic acid–Schiff; PAS). The expression of GPER was analyzed by immunohistochemistry and Western blot. GPER-mediated intracellular cAMP and calcium (Ca2+) signaling were measured immunoenzymatically or colorimetrically. Our data revealed changes in the distribution of polysaccharide content but not the protein and acid content in the cryptorchid testis. Polysaccharides seemed to be partially translocated from the interstitial compartment to the seminiferous tubule compartment. Moreover, the markedly decreased expression of GPER and GPER downstream molecules, cAMP and Ca2+, suggests their potential role in testis pathology. Increased estrogen levels in cryptorchid conditions may be linked to disturbed GPER signaling. We postulate that GPER is a prominent key player in testis development and function and may be used as a new biomarker of horse testis in health and disease.

## 1. Introduction

Testicular descent is an integral part of the male differentiation process. Testes migrate from the abdominal cavity to the scrotum. This process is essential for proper testis functioning and it consists of two phases: transabdominal and inguinoscrotal. The migration involves the regression of the cranial suspensory ligament and the development of the gubernaculums. The gubernaculum is subject to the superior hormonal regulation of androgens and insulin-like 3 (INSL3), which are absolutely required for testis descent [1]. In addition, the contribution of other hormones to testicular descent, such as Mullerian inhibiting substance (MIS) and estrogen, has been reported [2,3,4]. MIS has little effect on the gubernaculum but fetal and adult-type Leydig cells express the MIS receptor, which initiates testicular descent. In contrast, estrogens downregulate INSL3, resulting in maldescent [5]. Both the altered action of endogenous estrogen and the impact of environmental estrogen change the intratesticular androgen–estrogen ratio, thus disturbing testis descent [3]. It is well known that estrogen exerts its effects through the nuclear estrogen receptor (ER). In the rat gubernaculum, mRNA and proteins for ERα and ERβ have been detected [6]. In knockout ERα mice, gubernaculum growth retardation, atrophy, and even cryptorchid symptoms have been reported [7]. Findings by Zhang et al. [8] confirmed the expression of the G-protein-coupled membrane estrogen receptor (GPER) in the gubernaculum of mice. The authors demonstrated that diethylstilbestrol acted through GPER and additionally affected its expression. A number of studies showed that this noncanonical estrogen receptor is implicated in pleiotropic cellular responses to estrogen via non-genomic mechanisms (for review, see [9,10]). Unlike in genomic responses, where ERs following estrogen binding act as transcriptional factors and regulate gene transcription in the estrogen target tissues, the non-genomic cellular responses to estrogen start at the plasma membrane. This leads to rapid activation of a number of second messenger-triggered cytoplasmic signal transduction cascades [11]. At present, the expression of GPER and its effect on spermatogenesis and steroidogenesis, including GPER implication in the pathology of these processes, has been clearly demonstrated in humans, rodents, and boar [12,13,14,15,16,17]. In the horse reproductive system, the presence of GPER was studied in the spermatozoon neck [18]. Aside from the presence of ERs and their seasonal expression variations in horse spermatozoa, capacitation and acrosome reactions are postulated to be independent of ER-mediated action. In addition, estradiol weakly modulates spermatozoa motility and this effect is strictly associated with GPER and not with ERs. In stallions, and similarly in boar or bulls, a large amount of testicular estrogen, even higher than in females in estrus, is produced without an understood purpose [19]. In horses, estrogen synthesis is subject to seasonal regulation by incomplete spermatogenesis arrest [20]. The main site of estrogen production is present in Leydig cells, showing strong immunoreactivity for aromatase. The epididymis and prostate are additional sources of estrogen [21].

Epidemiological studies together with laboratory cryptorchidism models have shown a strong link between testis descent impairment and xenoestrogen exposure. Such exposure is also associated with increased estrogen levels and expression of ER in the undescended testes [22,23,24,25,26,27,28].

In horses, cryptorchidism is a common congenital malformation associated with the failure to descend by one or both testes into the scrotal sac. The major clinical consequences resulting from elevated temperatures in the undescended testes are impaired fertility and a significantly increased risk of testicular malignancy [29]. It is a well-known fact that cryptorchid testis is incapable of producing spermatozoa, or, in some cases when the retained testis is located in the inguinal canal close to the scrotum, rare production of spermatozoa has been noted [30,31]. No data on the fertilization abilities of such spermatozoa are available. The number of Sertoli cells determines the number of produced spermatozoa. In sexually immature males, the proliferation of Sertoli cells is finished when the blood–testis barrier is developed, which directly affects the quantity of spermatozoa produced in the adult. Spermatozoa quantity can be modulated only in experimental conditions, especially in males with spermatogenesis disturbances. In natural conditions, it does not occur [32].

In addition, in some cryptorchid horses, the testes are capable of producing testosterone, leading to undesirable sexual behaviors [33]. Of note, according to Cox et al. [34] and Claes et al. [35], in cryptorchid horses, testosterone levels vary greatly between individuals and fluctuate with age and season, making it unsuitable as a definitive test.

The mode of cryptorchidism inheritance remains unclear and appears to have a multifactorial etiology, and the phenomenon of high intratesticular estrogen levels is still an unresolved issue. The present studies were designed to provide additional insight into estrogen regulation and the role of GPER in healthy and cryptorchid horse testes. These comparative data can help to elucidate the molecular basis of cryptorchidism in horses.

## 2. Results

### 2.1. Topography and Biochemistry of Healthy and Cryptorchid Testes

**Figure 1:**
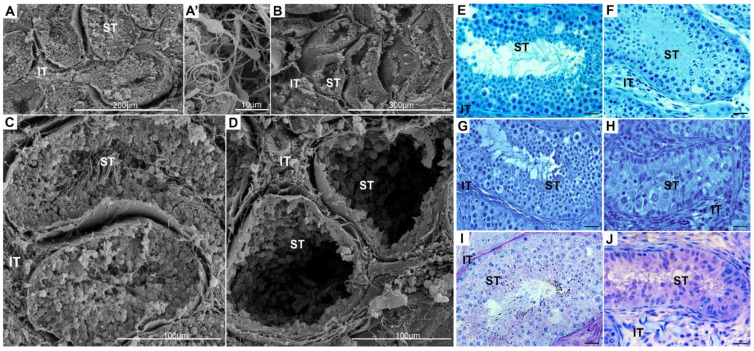
The general structure of healthy (**A**,**A’**,**C**,**E**,**G**,**I**) and cryptorchid (**B**,**D**,**F**,**H**,**J**) horse testicular tissues, respectively, which were used for analysis via scanning electron microscopy. Bars represent 1 µm. Histochemical staining of healthy and cryptorchid horse testes for the presence and localization of total protein content by naphthol blue black (NBB) (**E**,**F**), acidic content by toluidine blue O (TBO) (**G**,**H**), and polysaccharide content by PAS (**I**,**J**). Bar 20 µm. Histochemical staining was performed on three serial sections from each subject’s testis. IT—interstitial tissue, ST—seminiferous tubules.

The typical topography of a healthy testis with full spermatogenesis and spermatozoa visible in tubule lumen was compared to a cryptorchid testis with severe alterations (lack of several stages of seminiferous epithelium) using scanning electron microscopy (Figure 1A,A’,B–D).

The general biochemistry of the testes revealed typical histological features of healthy testis tissue, i.e., normal size of interstitial tissue. In the cryptorchid testis, it was overgrown; the observed increase was approx. 60% in comparison to the healthy testis. In addition, the cryptorchid seminiferous tubule diameter was decreased compared to healthy testes; the observed decrease was approx. 40% (Figure 1B,D). Histochemical staining for acid and protein content showed no marked differences between healthy and cryptorchid testes (Figure 1E–H). Both protein and acid content were distributed homogenously in the cells of the seminiferous tubule and the interstitial compartment. Of note, the lack of cells in the seminiferous tubules of cryptorchid testes coincided with a visible lack of proteins and acid in this testis compartment. However, polysaccharide content distribution demonstrated pronounced differences between healthy and cryptorchid testes (Figure 1I,J). In the healthy testes, the acidic content was concentrated in high amounts in the peritubular-myoid cells surrounding the seminiferous tubules and in the cells of the interstitial tissue, but there were small to moderate polysaccharide amounts in the cells of the seminiferous epithelium (Figure 1I). In the cryptorchid testes, polysaccharides were located mainly in the remaining cells of the seminiferous tubules, and a moderate amount was located in the interstitial tissue (Figure 1J).

### 2.2. GPER Protein Expression and Localization in Healthy and Cryptorchid Testes

**Figure 2:**
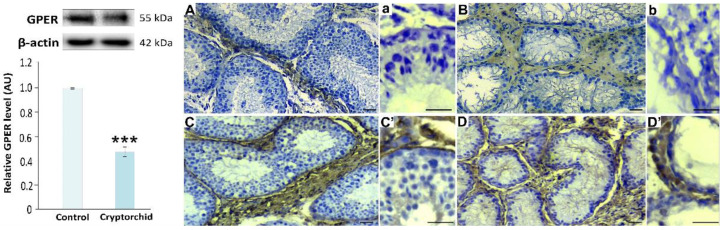
**Left panel.** Qualitative expression and relative expression (arbitrary units) of GPER in healthy (control) and cryptorchid horse testes. The relative amount of GPER normalized to β-actin. The relative intensity of bands from three separate analyses is expressed as means ± SD. Asterisks show significant differences between healthy and cryptorchid testes. Significant difference is denoted *** *p* < 0.001. **Right panel.** Representative microphotographs of immunohistochemical localization of GPER (**A**,**B**) and INSL3 (**C**,**D** and higher magnification **C’**, **D’** with positively immunostained individual Leydig cells) in healthy (**A**,**C**,**C’**) and cryptorchid (**B**,**D**,**D’**) horse testes. **a**,**b**—negative controls representative for GPER or INSL3 staining. Staining with DAB and counterstaining with hematoxylin. Bar 45 µm. Staining was performed at three serial sections from each animal.

A markedly decreased expression of GPER (*p* < 0.001) was revealed in cryptorchid testes when compared to healthy ones (Figure 2; left panel).

In healthy and cryptorchid testes, the expression of GPER was present exclusively in the Leydig cells (Figure 2A,B; right panel). However, the immunostaining was decreased in hyperplasic and hypertrophic Leydig cells in cryptorchid testes. Note that in horse testis, in contrast to human or rodent ones, the interstitial tissue is composed mainly of Leydig cells (please additionally see immunostaining for insulin-like protein 3 (INSL3)—marker of Leydig cells in healthy and cryptorchid horse testes; Figure 2C,C’,D,D’; right panel).

### 2.3. cAMP and Ca2+ Level in Healthy and Cryptorchid Testes

**Figure 3:**
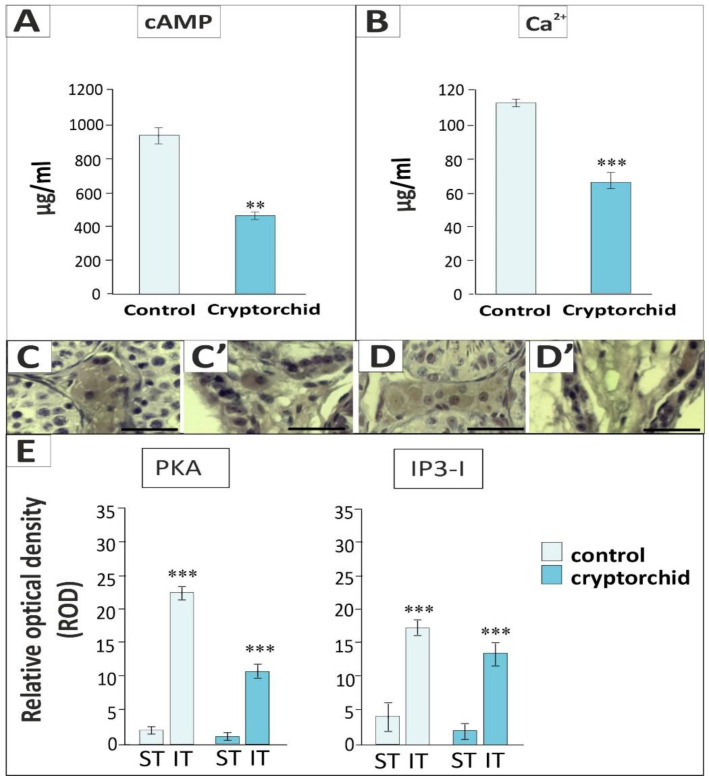
Upper panel: cAMP (**A**) and Ca2+ (**B**) levels; lower panel: representative microphotographs of immunohistochemical localization of PKA (**C**,**C’**), IP3 I (**D**,**D’**), and quantitative analysis of PKA or IP3 I immunosignals in the seminiferous tubules (ST) and interstitial tissues (IT) (**E**) of healthy (control) and cryptorchid horse testes. Data are expressed as means ± S.D. Significant differences between healthy and cryptorchid testes are denoted as ** *p* < 0.01 and *** *p* < 0.001. Analyses were performed in triplicate.

In cryptorchid testes, decreased levels of cAMP (*p* < 0.01) and Ca2+ (*p* < 0.001) were found when compared to the control (Figure 3A,B). These results were additionally confirmed by the decreased relative optical density (ROD) of the immunosignal for cAMP downstream enzyme protein kinase A (PKA) measured in the seminiferous tubules (*p* < 0.001) and interstitial tissue (*p* < 0.001) and for Ca2+ channel marker (IP3 I) measured in the seminiferous tubules (*p* < 0.001) and interstitial tissue (*p* < 0.001), respectively, in cryptorchid testes when compared to healthy ones (Figure 3C,C’,D,D’,E).

## 3. Discussion

It is still unclear how cryptorchidism can be prevented [36]. Moreover, recent studies by Murase et al. [37] revealed that, sometimes, the diagnosis can be challenging. The authors have uncovered undetectable serum MIS in cryptorchid horse testes. In this study, we used scanning electron microscopy analysis for further confirmation of the testicular tissue morphological status prior to subjecting the tissue to various biochemical and molecular analyses. Interestingly, in cryptorchid dogs, the right testis is more often affected than the left [38]. Our prior studies have demonstrated that senescence markers can be useful in the diagnosis of cryptorchidism in mixed-breed dogs [39]. In horses, as in dogs or humans, testis/testes located in the abdominal cavity can result in neoplasm development [40,41]. Here, for the first time, biochemical staining for protein, acid, and polysaccharide content in healthy and cryptorchid horse testes was performed. Both physiological and pathological tissue processes rely on the cell biochemical components that control cell functional specialization. Cellular components can serve to identify specific cell alterations or diseases [42,43,44,45]. The rich content of the biochemical substances produced by testicular somatic cells is crucial for germ and somatic cell function, especially for sperm physiology [46]. The polysaccharide content and distribution are affected in the cryptorchid testis. Cell division and an increase in glycosaminoglycans and hyaluronic acid levels cause caudal enlargement of the gubernaculum during the relative transabdominal movement of the testis, often referred to as the “swelling reaction” or “gubernacular out-growth” [47]. Glycoproteins are important in the regulation of the blood–testis barrier. Cyclic changes in glycoprotein composition and distribution occurring in spermatozoa and Sertoli cells are well described [48]. Our data confirm those reported by Su et al. [49], finding glycoprotein alterations in the pathological testis. In the cryptorchid testes, the remaining cells were overloaded with glycoproteins, which probably translocated from interstitial compartments and/or were overproduced in situ. Protein translocation and mislocalization is frequently reported in pathological tissues and cells [50]. In addition, any changes in the hormonal milieu can lead to morphological and biochemical tissue disturbances. Our earlier studies have revealed hormonal imbalances in horse cryptorchid testes, i.e., reduced testosterone synthesis and increased androgen aromatization [51]. Additionally, specific changes in testicular cell hormonal controls due to endogenous (or exogenous) factors also affect cell-to-cell and molecule-to-molecule interactions [52,53,54,55] that in turn affect the morpho-functional testis status.

Estrogens are necessary for maintaining the structural and functional integrity of the male reproductive tract [56]. Of note, the importance of estrogen in the male reproductive system’s development was confirmed by the report that diethylstilbestrol treatment during pregnancy induced, e.g., cryptorchidism, in mice and humans [57]. Furthermore, the concentration of estrogen in peripheral blood is typically low in the male, but ranges from 2 to 180 pg/mL in different species [58]. Exceptionally, in the horse, estrone sulfate is found as high as 2447 pg/mL and the estrone-sulfate concentration is 900 ng/mL in the testicular lymph, suggesting that intra-testicular estrogen levels can be high [57]. Estrogen concentrations are higher in the testicular vein and lymph than in the general circulation. Furthermore, in the reproductive tract, estrogen can reach relatively high concentrations. In addition, estrogens are abundant in semen, with concentrations ranging from 14 to 900 pg/mL in different species [59]. In the horse, estrone-sulfate is as high as 4000 pg/mL [58].

However, their role in testis descent and associated pathologies requires further in-depth investigation. Here, for the first time, we have confirmed that GPER is expressed in both healthy and cryptorchid horse testes. Of note, GPER positive expression was exclusively found in Leydig cells, which are the main source of testicular estrogens in horses. The responses of Leydig cells to elevated scrotal temperatures have been extensively studied in various models, including acute hyperthermia, experimental and spontaneous cryptorchidism, and heat acclimation [60]. In Leydig cells, following exposure to elevated testicular temperatures, changes in cell size and number, elevated levels of serum lutropin, low to normal serum testosterone levels, in vitro androgen hyper-responsiveness, loss of lutropin receptors, perturbed steroid biosynthesis, excessive accumulation of intracytoplasmic lipid droplets, deep nuclear invaginations, irregular outlines, and dilated smooth endoplasmic reticulum and mitochondria were reported [61]. Therefore, as revealed here, the biochemical and molecular alterations in the interstitial tissue of cryptorchid testes can result from the above-listed morphological and functional Leydig cell changes. Our present results add to a body of reports in a wide range of heat models establishing that the Leydig cell response occurs subsequently to damage of seminiferous tubules.

In addition to ERs, GPER is involved in maintaining estrogen levels in horse testes [62]. In fact, increased estrogen levels in a cryptorchid testis negatively affect GPER expression and signaling. Our findings show that GPER appears to be a key player in the regulation of estrogen-dependent lipid homeostasis and testosterone biosynthesis in Leydig cells [14,15]. Furthermore, our prior results have also shown the important role of GPER in regulating the number and physiology of the regulatory cells of the interstitial compartment telocytes, which contribute to maintaining lipid balance and steroidogenic function [62,63]. Based on the results of others, a lack of expression of GPER in the seminiferous tubule epithelium suggests a rather indirect modulation of the molecular mechanism involved in the maintenance of Sertoli cell numbers, normal testis development, and homeostasis [64,65]. In humans, GPER is expressed in peritubular-myoid cells, suggesting that estrogenic signaling via GPER is involved in the regulation of peritubular-myoid cells [66]. This is not the case in either healthy or cryptorchid horse testes. Similarly, some studies of the expression of GPER in germ cells suggest a role in modulating both the proliferation of spermatogonia and the physiological apoptosis regulating the number of spermatocytes and spermatids [17]. It seems possible that in healthy and cryptorchid horse testes, both of these processes are indirectly controlled by GPER.

Estradiol binds to GPER with a high affinity, while estrone and estriol have very low binding affinities [67]. Based on the previous studies in mouse and Atlantic croaker testes, the order of binding affinity of estrogens for the two canonical estrogen receptors α and β is similar to that of GPER (estradiol >> estrone = estriol) [68]. Furthermore, several environmental estrogens bind to GPER and activate the downstream signaling pathways, such as BPA, genistein, and nonylphenol [69]. In light of these facts, environmental hormonal factors are still an important consideration in cryptorchidism etiology. Decreased expression of GPER in the cryptorchid horse testis can be indicative of the alteration of GPER protein structure and/or amounts either by temperature factors and/or by bound xenoestrogens that further affect pathological testis structure and function. Currently, testicular GPER localization has been confirmed in a few mammalian species in normal and pathological conditions as well as in testicular primary cells in vitro and cell lines [17]. Our previous research shows expression of GPER in bank vole, mouse, boar, and human testes with leydigioma and GPER interactions with various proteins and lipids [14,15,62,63,70]. Interestingly, GPER has been found in several subcellular compartments, including the internal membrane of the endoplasmic reticulum, nucleus, and even in a chromatin-binding protein under certain circumstances [71], as confirmed in this study. Positive immunostaining in Leydig cell cytoplasm establishes the location of GPER in organelle structures. Of note, studies in GPER knockout mice have confirmed the role of GPER action on glucose and lipids in energy metabolism [72]. Specific studies using pharmacological GPER agonists and antagonists show that, in addition to acting on multiple intracellular pathways with specific actions on epidermal growth factor, extracellular signal-regulated kinases 1/2, phospholipase C, phospho-3-inositol, adenylyl cyclase, and Ca2+ immobilization, GPER is also able to mediate genomic responses [17]. Our data confirmed the action of cAMP and Ca2+ in GPER signal transmission. In the cryptorchid testis, decreased expression of GPER affected both Ca2+ and cAMP levels. These two universal second messengers impact various aspects of cell function, including hormone secretion, gene expression, stimulation of other messengers, muscle contraction, and metabolism. Due to both biochemical and molecular changes mainly in the interstitial tissue (which is the first physiological barrier protecting seminiferous tubules; [73]) in cryptorchid testes, an altered Ca2+ level and cAMP level is produced by this compartment. In fact, the largest somatic cells of the seminiferous epithelium, Sertoli cells, seem to be less sensitive to the temperature increase, still actively producing various molecules [74]. Changes in Ca2+ levels are obvious in the cryptorchid testis as the contractility of the gubernaculum during testis descent requires an influx of Ca2+ via dihydropyridine receptors [75]. Calcium ions are also required for steroidogenesis or spermatozoon acrosome reaction. However, both processes are altered in the cells of the cryptorchid testis as a consequence of decreased levels of Ca2+.

Additionally, the genomic effect of GPER via cAMP is included. Neural salient serine/arginine rich protein 1 (NSSR1) is a splicing factor expressed in germ cells and Sertoli cells. In mice, NSSR1 expression increases significantly during testes development, descent, and spermatogenic function [76]. In cryptorchid testes, dephosphorylated NSSR1 is significantly increased in association with cAMP response element binding protein (CREB) transcripts. CREB mainly acts as an inhibitor of cAMP-induced transcription through the acute regulation of trophic hormone-stimulated steroidogenesis and the acute steroidogenesis regulatory protein at the mRNA level in Leydig cells [77]. It is known that GPER can act as an autonomous receptor and can also interact with nuclear ERs based on studies performed on knockout mice and cultured cells [78]. However, the degree to which GPER acts autonomously depends on the cell type, differentiation status, and pathology, e.g., cell quiescence, proliferation, or cancer status. The time of cryptorchid testis retinues in the abdominal cavity may have an additional effect on the biochemical and molecular changes studied here [79].

**Figure 4:**
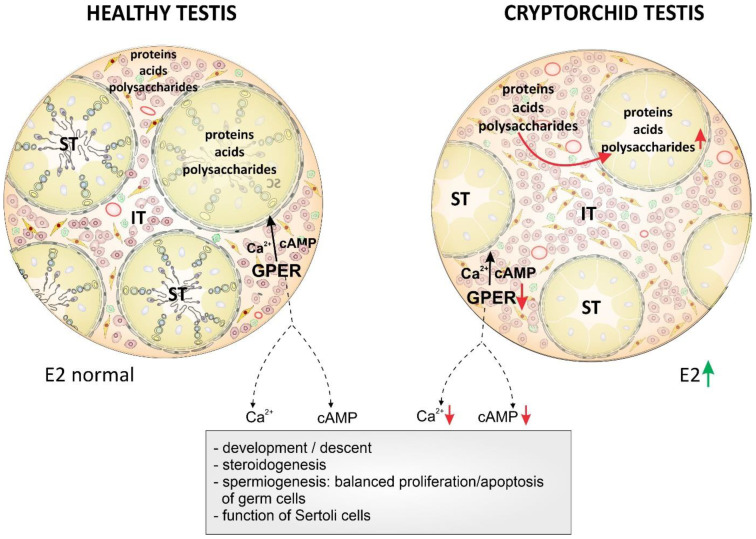
Schematic representation of the biochemical and molecular status of healthy and cryptorchid horse testes demonstrating the possible effects of GPER downstream pathways via cAMP and Ca2+ on the testes from development to maturity under both normal in healthy testes and the increased estrogen levels in cryptorchid testes.

In conclusion, a coexistence of (i) global biochemical changes in the testis compartments (referred to as polysaccharide distribution and amounts) and (ii) local molecular changes (decreased GPER expression in Leydig cells and decreased GPER downstream pathways via cAMP and Ca2+) in the cryptorchid horse testis is reported here (Figure 4).

Our results, considered with those by Zhang et al. [8], suggest that GPER in conjunction with estrogen exerts its effect on the gubernaculum, and GPER is an important molecule in cell–cell interactions in mature testis function (metabolic homeostasis and nutrition (based on polysaccharides and lipids) and steroid production. Perturbation of GPER action either by endogenous or exogenous factors affecting the estrogen microenvironment can contribute to pathological testis conditions including cryptorchidism in horses. Further studies are warranted to define the function of GPER in Leydig cells from fetus to adulthood in horse testes as well as to determine whether GPER can serve as a biomarker for the evaluation of normal or disturbed testis morphology and function.

## 4. Materials and Methods

### 4.1. Animals

Testes were obtained from 2–3-year-old unilaterally cryptorchid (*n* = 3; left testis in inguinal canal (*n* = 1) and right testis *n* = 2) Arab horses during open surgical castration (private studs in Warsaw and Krakow). Before castration, their libido was normal, indicating the presence of sufficient testosterone levels secreted by healthy testes.

Healthy testes (*n* = 2; archival material) were obtained from Arab horses that had been euthanized due to serious injury, e.g., fracture of the limb or spine during their moving around the stud, or fighting between horses.

Removed testes were either cut into small fragments for microscopic analyses or frozen in liquid nitrogen and stored at −80 °C for molecular analyses.

The use of animal tissues was approved by the Local Ethics Committee in Krakow (permission number: 105a/2015/08084).

### 4.2. Testis Topography—Scanning Electron Microscope (SEM)

Pieces of testicular tissue were fixed in a mixture of 2.5% formaldehyde with 2.5% glutaraldehyde in a 0.05 M cacodylate buffer (Sigma-Aldrich; Saint Louis, MO, USA; pH 7.2) for several days, washed three times in a 0.1 M sodium cacodylate buffer, and later dehydrated and subjected to critical-point drying. Specimens were then sputter-coated with gold and examined at an accelerating voltage of 20 kV or 10kV using a Hitachi S-4700 scanning electron microscope (Hitachi, Tokyo, Japan).

### 4.3. Testicular Tissue Processing and Histological Staining

Pieces of testicular tissue were embedded in Technovit 7100 (Kulzer, Germany). The tissue was fixed in Bouin’s solution (Sigma-Aldrich, Saint Louis, MO, USA) at 4 °C overnight and dehydrated in a graded ethanol series. Then, the tissue was preserved overnight in absolute ethanol and subsequently infiltrated with mixtures of absolute ethanol and Technovit. The samples were stored in pure Technovit, followed by polymerization of the resin with the addition of a hardener. Serial 5-µm-thick sections were prepared with the use of a rotary microtome (Microm, Adamas Instrumenten, Rhenen, The Netherlands). For general histology and for total protein, sections were stained with naphthol blue black (NBB) in acetic acid. Then, the slides were immersed briefly in acetic acid, air-dried, and mounted in Entellan synthetic resin (Merck, Darmstadt, Germany). For acid content, sections were stained with 0.1% toluidine blue O (TBO) and mounted in Entellan synthetic resin (Merck, Darmstadt, Germany). In order to detect insoluble polysaccharides, a periodic acid–Schiff (PAS) reaction was performed. Slides were oxidized in periodic acid and stained in Schiff’s reagent, then rinsed in potassium metabisulfite and mounted in Entellan synthetic resin (Merck, Darmstadt, Germany).

### 4.4. Immunohistochemistry

Testicular tissues were cut into 4-µm-thin sections. For antigen retrieval, endogenous peroxidase neutralization and blocking of non-specific binding sites were performed as described previously [80]. Thereafter, the sections were incubated overnight at 4 °C with a primary antibody: anti-GPER (Abcam; Cambridge UK; ref. no.39742), anti-INSL3 (Santa Cruz Biotechnology; Dallas, TX, USA ref. no sc-134587), anti-PKA II Antibody (Santa Cruz Biotechnology; Dallas, TX, USA; ref.no. sc-908), or anti-IP3 I (Sigma-Aldrich Saint Louis, MO, USA; ref.no. BT AP04603). Next, biotinylated goat anti-rabbit IgG (Vector Laboratories, Burlingame, CA, USA) and avidin-biotinylated horseradish peroxidase complex (ABC/HRP; Dako, Glostrup, Denmark) were applied in succession. The staining was developed using 3,3′-diaminobenzidine (DAB). Positive controls and negative controls in the absence of primary antibodies were performed for each immunostaining. Thereafter, sections were slightly counterstained with Mayer’s hematoxylin and mounted using DPX mounting media (Sigma-Aldrich; Saint Louis, MO, USA). Serial sections were examined with a Leica DMR microscope (Leica Microsystems, Wetzlar, Germany). The experiments were repeated three times on sections obtained from different animals.

### 4.5. Quantitative Analysis of Immunohistochemical Signal

The amount of PKA or IP3 I produced in the individual interstitial cells or seminiferous tubule cells was measured at ×100 magnification using ImageJ software https://imagej.nih.gov/ij/docs/intro.html (accessed on 5 January 2021). Measurements of freehand outlines drawn along the circumference of interstitial cell areas or seminiferous tubule areas were performed. On average, 60 areas of interstitial tissue or tubules were measured per slide. Relative optical density (ROD) of immunostaining insensitivities was determined for each animal testicular tissue and finally expressed as means ± SD.

### 4.6. Western Blot

Separation of protein preparations were performed by SDS-PAGE under reducing conditions and the transfer of proteins to polyvinylidene difluoride membranes. Nonspecific binding sites were blocked with non-fat dry milk containing Tween^®^ 20. Next, the membranes were incubated with anti-GPER antibody the same as for immunohistochemistry at 4 °C overnight, followed by a horseradish peroxidase-conjugated secondary antibody (Vector Laboratories, Burlingame, CA, USA) at room temperature. Proteins were detected by chemiluminescence and documented with a ChemiDocTM XRS+ System (Bio–Rad Laboratories, Hercules, CA, USA). The specificity of antibodies was assessed with the use of blocking peptides and/or positive controls (not shown). All immunoblots were stripped and re-probed with an anti-β-actin antibody (Sigma-Aldrich, Saint Louis, MO, USA; ref. no. A2228) as the loading control. The molecular weights of target proteins were estimated by reference to standard proteins (Sigma-Aldrich, Saint Louis, MO, USA). To obtain quantitative results, immunoblots were analyzed densitometrically (arbitrary units; AU) using the ImageLab software (Bio–Rad Laboratories, Hercules, CA, USA) by an independent observer.

### 4.7. cAMP Concentration Measurement

The amount of cAMP produced by healthy and cryptorchid horse testes was determined by Direct cAMP Elisa kit assay (Enzo, Life Sciences, AG, Lausen, Switzerland), according to the manufacturer’s instructions for the acetylated assay. The sensitivity of the assay was 0.037 pmol/mL. The cAMP concentrations were calculated as pmol/mL.

Three independent experiments were performed, each in triplicate.

### 4.8. Ca2+ Concentration Measurement

The amount of Ca2+ produced by healthy and cryptorchid horse testes was determined by ab102505 Calcium Assay Kit (Colorimetric) (Abcam; Cambridge, UK) according to the manufacturer’s instructions. The Ca2+ concentrations were calculated as ng/mL (OD = 575 nm). Three independent experiments were performed, each in triplicate.

### 4.9. Statistics

Each variable was tested using the Shapiro–Wilk W-test for normality. The homogeneity of variance was assessed with Levene’s test. The distribution of the variables was normal and the values were homogeneous in variance. All statistical analyses were performed using a one-way analysis of variance (ANOVA) followed by Tukey’s post hoc comparison test to determine which values differed significantly from controls. The analysis was performed using Statistica software (StatSoft, Tulsa, OK, USA). Data were presented as a mean ± SD. Data were considered statistically significant at *p* < 0.05. All experimental measurements were performed in triplicate from material derived from different animals.

## Data Availability

Data will be presented on request.
